# Selective detections of single-viruses using solid-state nanopores

**DOI:** 10.1038/s41598-018-34665-4

**Published:** 2018-11-02

**Authors:** Akihide Arima, Makusu Tsutsui, Ilva Hanun Harlisa, Takeshi Yoshida, Masayoshi Tanaka, Kazumichi Yokota, Wataru Tonomura, Masateru Taniguchi, Mina Okochi, Takashi Washio, Tomoji Kawai

**Affiliations:** 1 The Institute of Scientific and Industrial Research, Osaka University, 8-1 Mihogaoka, Ibaraki, Osaka 567-0047 Japan; 2 Department of Chemical Science and Engineering, School of Materials and Chemical Technology, Tokyo Institute of Technology, 2-12-1, O-okayama, Meguro-ku, Tokyo 152-8552 Japan

## Abstract

Rapid diagnosis of flu before symptom onsets can revolutionize our health through diminishing a risk for serious complication as well as preventing infectious disease outbreak. Sensor sensitivity and selectivity are key to accomplish this goal as the number of virus is quite small at the early stage of infection. Here we report on label-free electrical diagnostics of influenza based on nanopore analytics that distinguishes individual virions by their distinct physical features. We accomplish selective resistive-pulse sensing of single flu virus having negative surface charges in a physiological media by exploiting electroosmotic flow to filter contaminants at the Si_3_N_4_ pore orifice. We demonstrate identifications of allotypes with 68% accuracy at the single-virus level via pattern classifications of the ionic current signatures. We also show that this discriminability becomes >95% under a binomial distribution theorem by ensembling the pulse data of >20 virions. This simple mechanism is versatile for point-of-care tests of a wide range of flu types.

## Introduction

Influenza is a highly contagious respiratory disease of worldwide concern from individual to global perspectives: it annually causes millions of infections with a potential risk to serious outbreak due to the high mutability of flu virus^1–4^. In contrast, there is no effective way except subsidiary passive vaccination to negate a detrimental impact of the infectious pathogen ubiquitous in the environment^5–7^. A means for rapid diagnosis of the infectious disease has thus been explored as one of the strategies for preventing seasonal epidemic to possible pandemic through enabling medication at very early stage of infection^8–10^. Despite the continued progress in the performance of commercial immunosensors^10–12^, however, the sensitivity is still not high enough especially for the existing allotypes^13^ nor novel strains lacking antigenicity to the existing antibodies^14^ for diagnosis before symptom onsets., immunosensing and genetic approaches are considered as promising strategies. On the other side, while a genetic approach such as reverse transcription polymerase chain reaction^15,16^ is a versatile approach capable of identifying essentially any virus species, it relies on time consuming amplification processes with expensive facilities requiring expertise for the operation and hence not for prompt screening^17^. Considering that modern global society creates ever-increasing opportunities for an outbreak, it is thus of urgent importance to find sensitive sensor platforms viable for multiplex detections of the nanoscale bioparticles^18^.

To this end, we herein report on a novel sensor concept capable of discriminating various types of influenza virus in label-free fashion by their distinct particle properties. We utilized a nanopore technology^19–22^ for single-virus detections in a physiological environment. While it was demonstrated previously that viruses of different sizes can be discriminated by the height of resistive pulses using conventional long fluidic channels, the method is anticipated to be not applicable for distinguishing the essentially equi-sized viral nanoparticles of influenza types. The nanopores in the present study were therefore designed to have low thickness-to-diameter aspect-ratio structure^23–28^ so as to render additional sensitivity to the particle shape and surface charges whereby provide resistive pulses holding complex set of information concerning not only the nanoparticle volume but multiple physical properties of the intact viral particles. Although this would in general complicates the physical interpretation of the electrical signals wherein numerical simulations often play central roles to elucidate the electrokinetic phenomena^29,30^, we employed a machine-learning-driven pattern-analysis of the electrical signatures for rapid detection and simultaneous subtype differentiation with an ultimate sensitivity of single-particle discriminations.

## Results

### Single-virus detections using a solid-state nanopore

**Figure 1:**
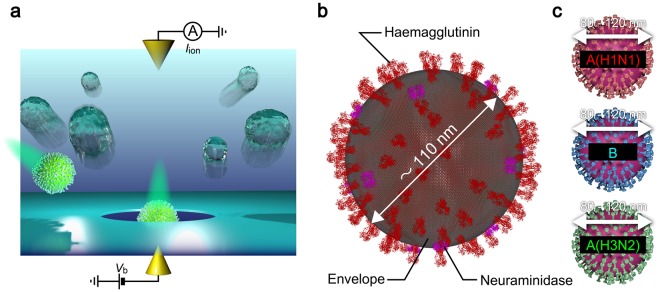
Single-influenza-virion detections using a solid-state nanopore. (**a**) Schematic illustration depicting nanopore measurements. Individual influenza virions in chorioallantoic fluid were passed through a Si_3_N_4_ nanopore via electrophoresis under the applied voltage *V*_b_ and associated resistive pulses were recorded by tracing a temporal change in the cross-membrane ionic current *I*_ion_. (**b**) Influenza virion consisting of a spherical capsid covered with envelope and protein spikes such as haemagglutinin and neuraminidase protruding from the surface. This figure was created using the protein structure of the haemagglutinin^32^ (https://www.rcsb.org/structure/1ru7) and neuraminidase^33^ (https://www.rcsb.org/structure/5hun) from the Research Collaboratory for Structural Bioinformatics (RCSB) Protein Data Bank (PDB) website. (**c**) Three types of influenza viruses employed for nanopore sensing (Top: A(H1N1); middle: B; bottom: A(H3N2)).

**Figure 2:**
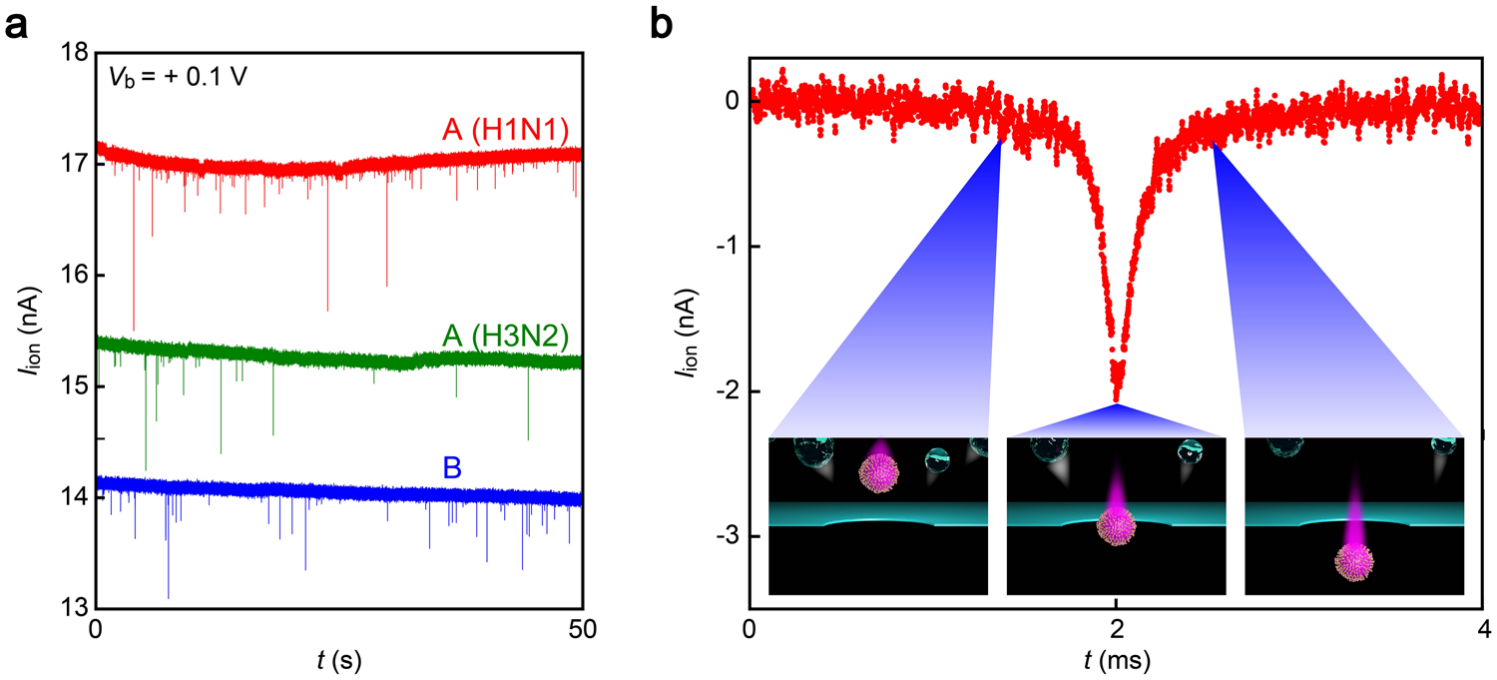
Virion-derived resistive pulses. (**a**) Ionic current (*I*_ion_) traces recorded in chorioallantoic fluid containing A(H1N1) (red), A(H3N2) (green), or B (blue) using 300 nm-sized Si_3_N_4_ nanopore under the bias dc voltage *V*_b_ = +0.1 V. Each spike-like *I*_ion_ change signifies electrophoretic translocation of single-virion through the pore channel. (**b**) A magnified view of a resistive pulse. The open pore current is offset to zero. Insets describe the viral motion upon transit through the conduit. Contaminants in the chorioallantoic solution depicted as transparent particles are repelled from the nanopore via electroosmotic flow during the virus sensing under the applied positive voltage.

Our device consists of a hole of diameter 300 nm sculpted in a 50 nm thick Si_3_N_4_ membrane on a Si wafer (Fig. 1a). We utilized the solid-state nanopores for single-virus detections of influenza A(H1N1), A(H3N2), and B. These strains have common size and spherical shapes but different surface proteins such as haemagglutinin and neuraminidase^31^. Measuring the ionic current *I*_ion_ through the solid-state nanopore whose one side filled with chick chorioallantoic fluid containing flu virus (Fig. 1b,c)^32,33^ and the other side with phosphate buffer saline (PBS) under the applied dc voltage *V*_b_ of +0.1 V (Fig. S1), we observed transient current drops each signifying temporal exclusion of ions inside the conduit by an individual virus upon translocation (Fig. 2a,b; see also Supplementary Information Fig. S2)^34^. In contrast, inverting the sign to –0.1 V resulted in fluctuations of the open pore current with no resistive pulse signals. This bias polarity dependence, which was confirmed in all the three types of viruses measured, suggests electrophoretic capture of the negatively-charged viruses at the pH conditions under positive *V*_b_^35^.

### Electroosmotic filter effects for selective detections of influenza viruses

Here, special care was taken to assure that the *I*_ion_ spikes are of viral particles of concern as the chick chorioallantoic fluid is a mixture of various ingredients such as mucins^36^. For this, we conducted a control in a virus-free chorioallantoic solution (Fig. S3). As a result, we found no resistive pulses under *V*_b_ = +0.1 V whereby unambiguously proving that the current spike signals stem from translocation of influenza viruses through a nanopore. Meanwhile, the large *I*_ion_ fluctuations at negative *V*_b_ also seen in the control was attributed to contributions of the electroosmotic flow in Si_3_N_4_ nanopores having negative native charges on the wall surface (zeta potential of – 42 mV at pH 7.6 as obtained using a zeta sizer (Malvern) that agrees with previous reports) to drag weakly charged biomolecules and particles into the channel (Fig. S4). This in turn manifests an intimate role of the field-induced fluid flow to effectively filter non-virus objects during the virus detections, which we found valid also in other biological media such as human saliva (Fig. S5) thereby proving the applicability of the technique as influenza diagnostic tools^37^. It is emphasized that the present method requires no laborious pretreatment such as ultracentrifugation but only a simple filtration using a commercial membrane filter (see Methods).

### Single-virus identifications by one ionic current pulse

Having ensured that the resistive pulses were associated with single-virus translocation through the nanopore, we examined discriminations of the influenza types by investigating the difference in the spike waveforms. This is *a priori* expected to be a formidable task to achieve as the viruses have spherical motif of similar sizes around 100 nm irrespective of genetic variations^38,39^. In fact, we observed little difference in the resistive pulse height, a feature reflecting the volume of the pathogenic bioparticles, among the three viruses (Fig. S6). However, unlike conventional Coulter counters wherein channels are designed to optimize the sensitivity for measuring particle size through making the membrane thickness *L* relatively larger than the pore diameter *d*, our nanopores have a low *L*/*d* aspect ratio structure that renders better spatial resolution to analyse shape of analytes and additional sensitivity to the surface charge status^40,41^. Moreover, it possesses a wide sensing zone extending by distance 300 nm from the channel as shown by the multiphysics simulations (Fig. S2) that provides a unique capability to electrically sense dynamic motions of the electrophoretically-drawn viral particles at the nanopore orifice^42^. The ionic current spike patterns, therefore, comprise a wealth of information about not only the particle size and retention time in a channel but also its shape and capture dynamics that would differentiate the three kinds of influenza measured here^40,41^.

**Figure 3:**
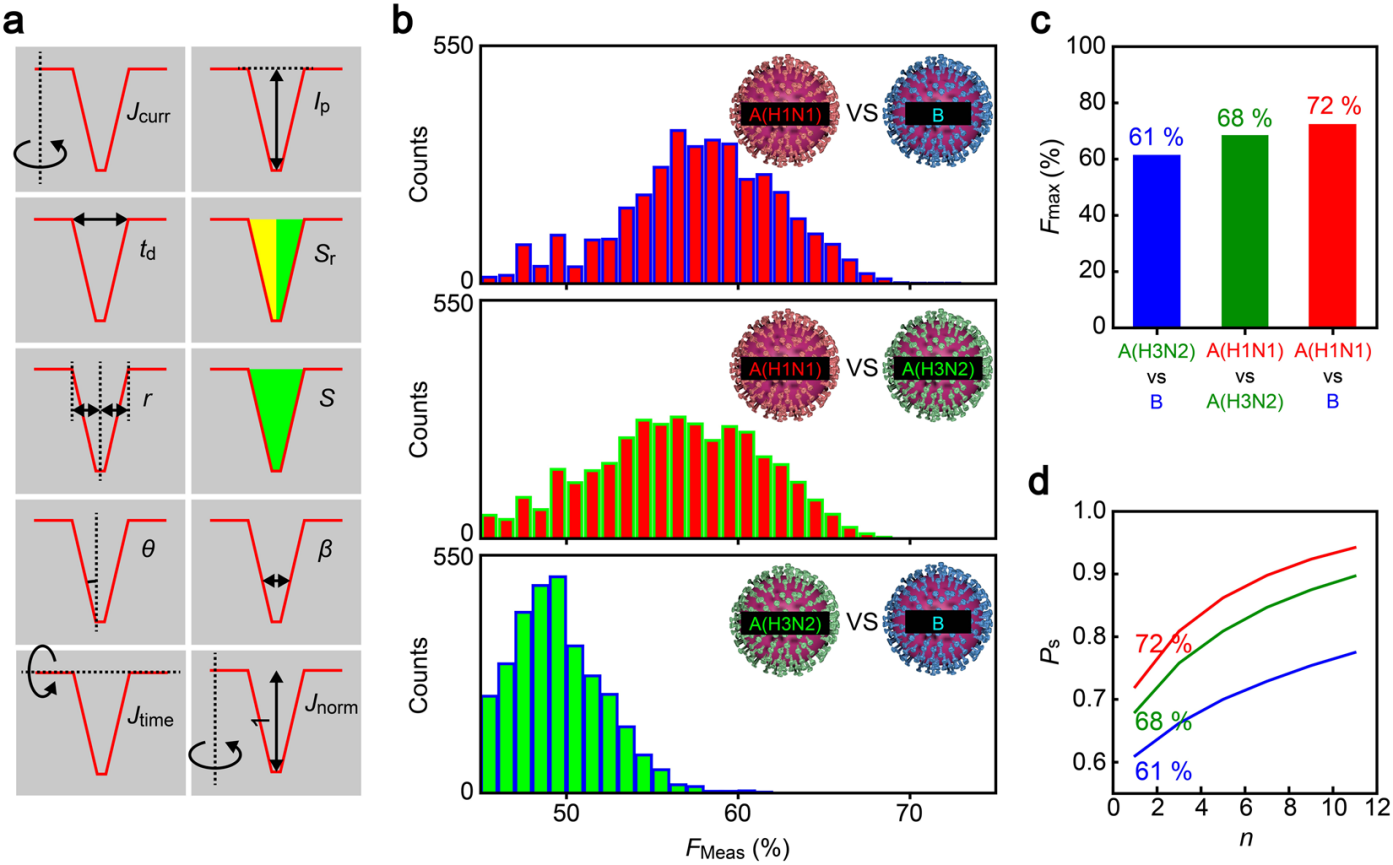
Machine learning based single-pulse identifications. (**a**) Resistive pulse features used for single-virus discriminations. (**b**) F-measure score, *F*_meas_, deduced for cases of distinguishing A(H1N1) and B (top); A(H1N1) and A(H3N2) (middle); and A(H3N2) and B (bottom). The distributions are constructed with 4260 *F*_meas_ data output by 71 classifiers and 60 feature vectors utilized in the single-pulse analysis. (**c**) The highest *F*_meas_, *F*_max_. (**d**) Dependence of the influenza type discriminability *P*_s_ on the number of detected virions *n*. Color coding is the same as that in (**c**).

In order for the single-virus diagnosis, we employed a machine learning approach to identify characteristic single-virus signatures in the fine corrugations of ionic current pulses^43,44^. Specifically, we used the Rotation Forest^45^ ensemble method in WEKA (The Waikato Environment for Knowledge Analysis) workbench^46^. The algorithm randomly selects several feature parameters of an *I*_ion_ spike among the predefined ten factors (Fig. 3a; see also Fig. S7–9 and Table S1) and data points to create vectors in feature space for teaching classifiers, which then serve as decision trees to judge whether resistive pulses are of A(H1N1), A(H3N2), or B. Using a ten-fold cross-validation method, the virus discriminability was assessed in terms of F-measure score *F*_meas_ = 2*P*_pre_*P*_rec_/(*P*_pre_ + *P*_rec_), where *P*_pre_ and *P*_rec_ are the precision and recall calculated through the number of true-positive, false-positive, and false-negative outputs. As a result, we accomplished discrimination of A(H1N1) vs B, A(H1N1) vs A(H3N2), and A(H3N2) vs B by a single resistive pulse with accuracy of 72%, 68%, and 61%, respectively (Fig. 3b,c). We emphasize that this discriminability was assessed from the resistive pulse data of the three types of viruses obtained with the same nanopore chip to avoid any influence of pore-to-pore variance, although such effects were anticipated to be minor as remarked by the negligible variation in the channel size (Fig. S10) along with little difference in the ionic current spike patterns among the devices used (Fig. S11).

### Digital diagnosis of influenza

We emphasize that the above discriminability is for single pulse analysis; in practice, the reliability of disease diagnosis can be increased by the number of virus-derived ionic spikes collected. For example, it is naturally anticipated that patients normally catch only one type of flu. The problem is then simplified to follow the binominal theorem that predicts the spike-number-dependent recall *P*_s_ to be >94% when *n* >11 for verifying A(H1N1) or B infections when allowing a majority decision among the classifiers to give eventual diagnosis. Furthermore, even if the test includes allotype detections, it can be implemented at discriminability better than 78% as deduced from *P*_rec_ in the most difficult case of distinguishing A(H3N2) from B (Fig. 3d). Although it is not straightforward to directly compare the performance with the commercial immunosensors, the nanopore diagnostics would be capable of providing qualitative decisions with a practically-viable accuracy backed by quantitative *P*_rec_ values to estimate the output reliability.

### Physics underlying the virus type discriminability

**Figure 4:**
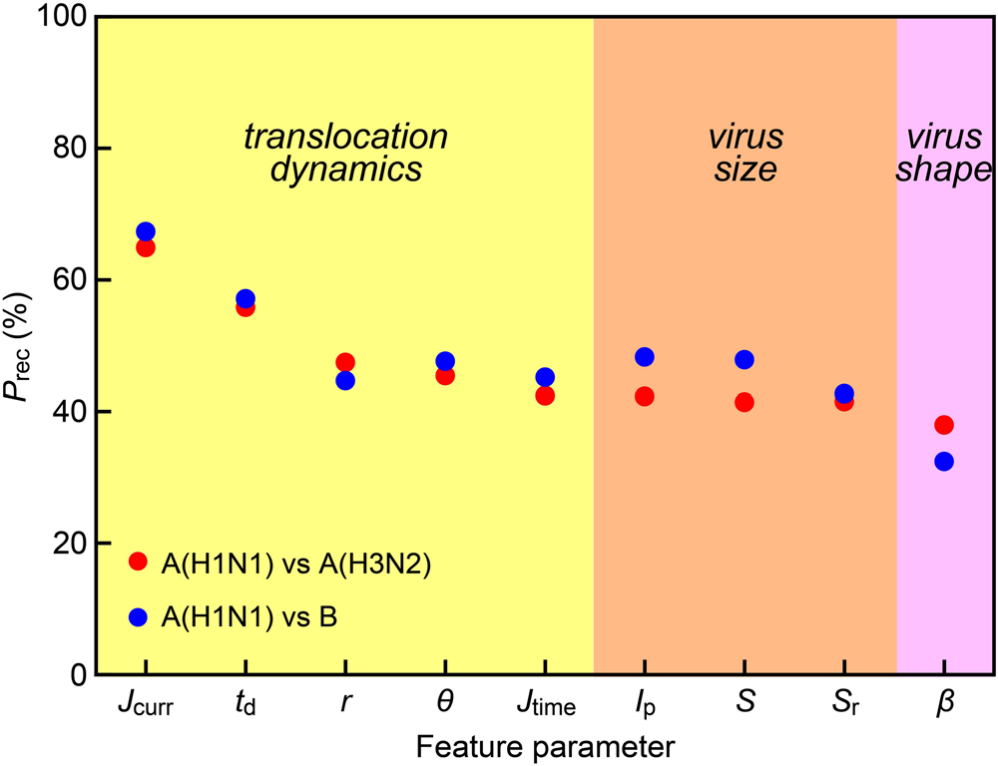
Dissecting resistive-pulses to elucidate physical features of single-viruses. Recall *P*_rec_ to discriminate A(H1N1) and A(H3N2) (red) or A(H1N1) and B (blue) plotted as a function of the feature parameters. Each feature reflects distinct physical characteristics of single-virus translocated through a nanopore, *i*.*e*. the translocation dynamics (yellow), the size (orange), and the shape (pink). The higher *P*_rec_ with the dynamics-reflecting features suggests that the machine learning algorithm discriminated influenza types according to a difference in their surface charge densities rather than the morphologies.

It is of interest to clarify what enabled the algorithm to identify the virus types. We investigate this by deducing *P*_rec_ based on non-parametric probability distributions of combinations of specific feature parameters among the choices in those used in the single-virus identifications (Fig. 3a). Interestingly, the statistical analysis yielded relatively low *P*_rec_ when employing the pulse height *I*_p_ or bluntness *β*, the indices of which are known to reflect the size and shape of a particle, respectively (Fig. 4). Meanwhile, high-*P*_rec_ discrimination was attained with the pulse width *t*_d_ that denotes the electrophoretic speed of particles passing through a nanopore. It is thus the translocation dynamics rather than the morphological aspects that mostly contributed to differentiate the viral particles. In fact, comparison of *t*_d_ distributions revealed that the three viruses possess different surface charge densities in order A(H1N1) > A(H3N2) ~ B, which is consistent to the relatively low *F*_meas_ acquired for the case of A(H3N2) vs B (Fig. S12–S13). Physically, the shorter translocation duration of A(H1N1) is ascribable to larger net negative surface charges, due presumably to less positive haemagglutinin units at the envelope compared to those of the other two types^34,47^, that lead to higher electrophoretic mobility.

The above discussion indicates that modifying the nanopore sensor to render larger difference in *t*_d_ would provide better discriminability of the viruses. In this context, nanopore surface functionalization may be a promising approach that can slow-down the bioparticle translocation speed^48,49^ thereby enabling more accurate estimations of the event lifetime. Such strategy has also proven to be useful for the particle discriminations when utilizing recognition probes such as DNA^50^ and oligopeptides^51^ having specific affinity to molecules of concern, which retard the translocation motions of only particular analytes through the intermolecular interactions at the channel wall surface.

Validity of the feature-based classification for the virus identifications was further inspected by applying the single-pulse analysis to various analytes. For equi-sized polystyrene beads with different surface modifications, which is similar to the case of influenza diagnosis, we found dynamics-reflected parameters like *t*_d_ as effective for differentiation (Figs S14 and S15). On the other hand, when there was a size variance in the polymer spheres, on the other hand, *I*_p_ became the primary factor to discern the particles as expected (Fig. S15). In addition, even when distinguishing two bacteria having similar size and surface charge status, the pattern-analysis could still find a small difference in the shape when exploiting a low-aspect-ratio pore sensors wherein *β* played an increasing role for the discrimination (Fig. S16). This extensive capability would enable multi-detections of pathogens having potential risk for pneumonia^52^ along with influenza viruses.

## Conclusion

We herein reported a proof-of-concept demonstration of viral infection diagnosis using solid-state nanopores. We found an important role of the electroosmotic flow to impede impurity molecules in physiological media from entering Si_3_N_4_ pore channel whereby accomplished selective electrical detections of individual influenza virions. We demonstrated single-virus identifications to digital diagnosis of influenza including allotypes with high accuracy via resistive pulse measurements backed by a machine-learning-driven pattern recognition, wherein particle-dynamics-related ionic spike features were particularly effective for the viral discriminations revealing nanopore-discernible difference in the surface protein-derived charge densities on the different types of flu viruses. The single-virus diagnostic capability of the present method may be useful for point-of-care testing at very early stages of infection that can contribute to prevent disease outspread by enabling clinical treatments before symptom onsets.

## Methods

### Influenza virus multiplication

Influenza A(H1N1) (A/PR/8/34 (American Culture Collection, VR5)), Influenza A(H3N2) (A/Hong Kong/8/68 (American Type Culture Collection, VR-544)), and Influenza B (B/Lee/40 (American Type Culture Collection, VR-1535) viruses were inoculated in embryonated eggs. Specifically, we incubated chick eggs at 37 degrees Celsius and 60% humidity for 10 to 11 days. Subsequently, we inoculated one of the influenza viruses into each egg and further incubated for 3 days for multiplication. After that, we extracted chorioallantoic fluid at chorioallantois containing the multiplicated viruses. Before using for nanopore measurements, the virus solution was filtered at 450 nm (Millex-HV 0.45 μm; Millipore Co.) using a syringe. All the experiments using Influenza virus including inoculation, harvest, and nanopore measurements were conducted in a BSL-2 laboratory and it is confirmed by the Institutional Biosafety Committee.

### Nanopore fabrications

A Si_3_N_4_ layer coated Si wafer was used as a substrate. We first partially removed the Si_3_N_4_ layer by a reactive ion etching (RIE) with CF_4_ etchant gas. The exposed Si surface was then immersed in KOH solution made by dissolving KOH pellets (Wako Co., Japan) in ultrapure water (Millipore Co.) at 25% concentration and heated to 100 degrees Celsius for wet etching. As a result, a deep trench was formed with a 50 nm thick Si_3_N_4_ membrane created at the bottom. On the membrane, we delineated a 300 nm diameter circle in ZEP520A resist (Zeon Co.) by an electron beam lithography. After development, the remaining resist layer was used as a mask to sculpt a 300 nm-sized nanopore by an RIE process. Finally, the substrate was kept in *N*,*N*-dimethylformamide (Wako Co., Japan) overnight to dissolve the residual resist followed by rinse in ethanol and acetone.

### Ionic current measurements

In prior to the ionic current measurements, two pieces of polydimethylsiloxane (PDMS) blocks were adhered on both sides of a nanopore chip immediately after activating their surface via O_2_ plasma treatment. On the PDMS blocks, there were microchannels formed for flowing solution through inlet and outlet holes to fill the pore. Single-virus detections were implemented by using two Ag/AgCl electrodes to measure the ionic current *I*_ion_ through the nanopore. Here, a battery-driven potentiostat was used to apply the dc voltage *V*_b_. Meanwhile, the output current was amplified by a home-made preamplifier having a bandwidth wider than 1 MHz and recorded by digitizing (NI-5922, National Instruments Co.) and data-streaming (NI HDD-8264, National Instruments Co.) at 1 MHz sampling rate. Upon starting the measurements, we filled one side of a nanopore (where deep trench was formed) with ×0.25 PBS and the other side with the chorioallantoic solution containing Influenza virus of either A(H1N1), A(H3N2), or B. Clogging of a pore presumably by aggregated virus colloids was occurred in occasion. When this happened, we flown the virus solution to release the trapped particle (Note that reversing the voltage polarity to remove the clogged particle does not work well as it will induce electroosmotic flow to hydrodynamically drawn the weakly-charged ingredients like mucin in chorioallantoic fluid).

### Resistive pulse extraction

Resistive pulses were extracted from the ionic current traces as follows. Firstly, the moving open pore current was offsetted to zero by linearly fitting the partial *I*_ion_ curves of every 0.5 seconds followed by subtraction of the linear component from the raw data. Then, resistive pulses were extracted by searching for the point whereat the fluctuations of the ionic current increases (decreases) above (below) 5*σ* to find pulse onset (offset). Furthermore, the peak regions were expanded by 0.256 ms at both sides of the onset and offset whereby defining the regions of ionic current blockade events. These processes were implemented under a home-built computer program.

### Machine learning approach

Single-virus discriminations were performed by pattern-analyze individual ionic current spikes using classification algorithms of machine learning. For this, we used 60 different feature vectors which are different combinations of features and feature sub-vectors. They are 10 individual features of the ionic current pulse waveforms (*J*_curr_, *t*_d_, *r*, *θ*, *J*_time_, *J*_norm_, *I*_p_, *S*_r_, *S* and *β*) depicted in Fig. 3a, a current sub-vector *h*_v_ that consists of a sequence of the average original and normalized currents at *k* time sections (*k* = 8, 16, 32, 64, or 128) and a time sub-vector *t*_v_ defined as a sequence of the average time difference between the beginning and the peak of a pulse (*t*_L_) and that between the peak and the ending of the pulse (*t*_R_) at *l* current levels (*l* = 8, 16, 32, or 64). Pattern analysis of ionic current spikes of A(H1N1), A(H3N2), and B was exhibited on basis of the thus acquired dataset using The Waikato Environment for Knowledge Analysis (Weka) machine learning workbench with 71 Rotation Forest ensembles where each applies a distinct base classifier such as naïve Bayes models. In this supervised learning, 142 pulses of each virus, either A(H1N1), A(H3N2), or B were used as teacher data to judge the other 48 spikes. The output was further evaluated to deduce F-Measure score *F*_meas_ = 2*P*_pre_*P*_rec_/(*P*_pre_ + *P*_rec_), where *P*_pre_ and *P*_rec_ are the precision and recall calculated through TP/(TP + FP) and TP/(TP + FN), respectively, with TP, FP, FN denoting respectively the number of true-positive, false-positive, false-negative cases.

On the other hand, statistical estimations of the respective numbers of A(H1N1), A(H3N2), and B were conducted by applying non-parametric probability density estimation of each virus in feature spaces where every feature space is formed by a pairwise combination of distinct features of the ionic current waveforms. Specifically, we chose the aforementioned *J*_curr_, *t*_d_, *r*, *θ*, *J*_time_, *I*_p_, *S*_r_, *S* and *β*. Once the distribution of the pairs of the pulses in the feature space is given, the distribution is approximated by a weighted combination of the non-parametric probability densities learnt beforehand for the three virus species. The weights optimally approximating the distribution are learnt by using the expectation–maximization (EM) algorithm. The weights which provide optimal approximation are proportional to the respective numbers of the three virus species. The precision of the estimated numbers of the three virus species are represented by ${P}_{rec}=\{1-\sum _{{\rm{i}}=1}^{3}({{\rm{n}}}_{{\rm{i}}}/{\rm{N}})(|{{\rm{n}}}_{{\rm{i}}}-{\hat{{\rm{n}}}}_{{\rm{i}}}|/{{\rm{n}}}_{{\rm{i}}})\}$ where *n*_*i*_, ${\hat{n}}_{i}$ and *N* are the true number of the *i*-th species’ pulses, its estimation ${\hat{n}}_{i}={w}_{i}N$ by the estimated weight *w*_*i*_ and the total number of the virus pulses.

## Electronic supplementary material

Supplementary Information

## Data Availability

All data generated or analysed during this study are included in this published article and its Supplementary Information files.
