# Supplementary material for: Selective detections of single-viruses using solid-state nanopores
Source: Sci Rep. 2018 Nov 2;8:16305. doi: 10.1038/s41598-018-34665-4 (PMC6214978; doi:10.1038/s41598-018-34665-4)
Supplement: Supplementary file 1 — Supplementary Information [file 41598_2018_34665_MOESM1_ESM.pdf]

Supplementary information for

Selective detections of single-viruses using solid-state nanopores

Akihide Arima, Makusu Tsutsui, Ilva Hanun Harlisa, Takeshi Yoshida, Masayoshi Tanaka, Kazumichi Yokota, Wataru Tonomura, Masateru Taniguchi, Mina Okochi, Takashi Washio & Tomoji Kawai

The Supplementary Information includes:

1. Supplementary Figures (Figs. S1-S16)
2. Supplementary Table (Tab. S1)

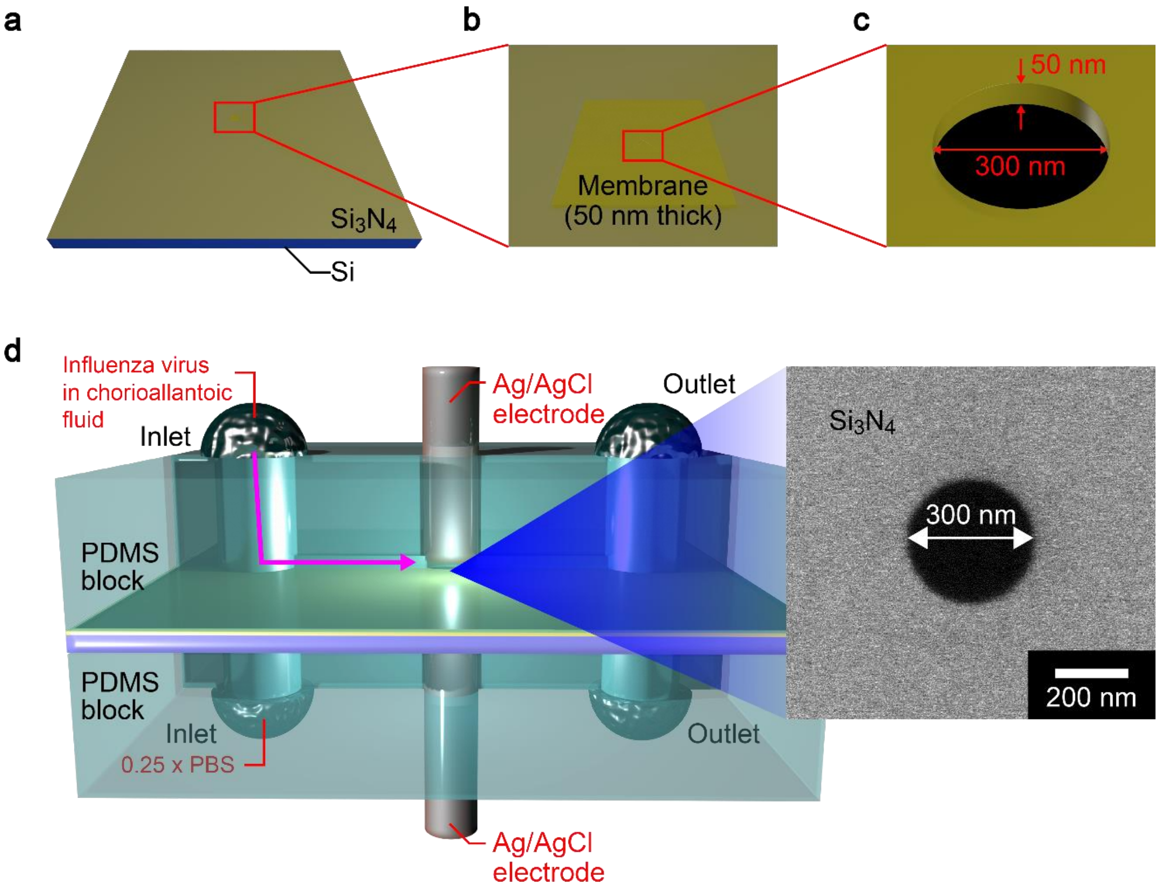

Figure S1. Nanopore sensor used for single-virus detections. **a-c,** Si wafer coated with 50 nm thick Si_3N_4 layers at the both sides by a low-pressure chemical vapor deposition was used as a substrate (a) whose Si was partially removed by wet etching in KOH aq. from one side so as to form a Si_3N_4 membrane of 100 μm x 100 μm size (b). In the membrane, a nanopore of diameter 300 nm was sculpted by electron-beam lithography and reactive ion etching methods (c). **d,** The thus fabricated nanopore was sealed by adhering polydimethylsiloxane (PDMS) blocks at the both sides. On each block, three holes are drilled: two for inlet and outlet to inject test solution and one for inserting Ag/AgCl electrode for the ionic current measurement. Here, virus-containing chorioallantoic fluid is added from one side of the nanopore while the other side is filled with 0.25 x PBS buffer. At the right displays a scanning electron micrograph of the nanopore.

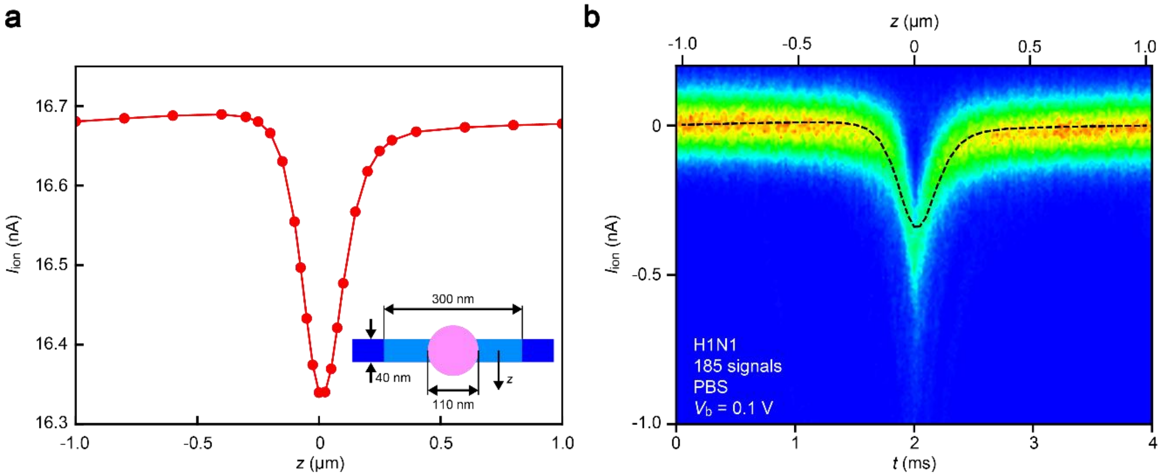

Figure S2. Simulation of resistive pulse. a, Ionic current blockade by a nanoparticle of diameter 110 nm mimicking an influenza virus translocating through a 300 nm-sized nanopore calculated using COMSOL under V_b = + 0.1 V. Ion concentrations are set according to the experimental condition wherein chorioallantoic solution is injected from one side and 0.25 x PBS on the other side. **b,** Two-dimensional histogram constructed with 185 signals obtained for A(H1N1) viruses under V_b = + 0.1 V. Dashed line is the simulated resistive pulse displayed in (a) that shows good agreement with the measurement results. Here, the theoretical pulse was deformed in the lateral direction to fit the experimental spikes.

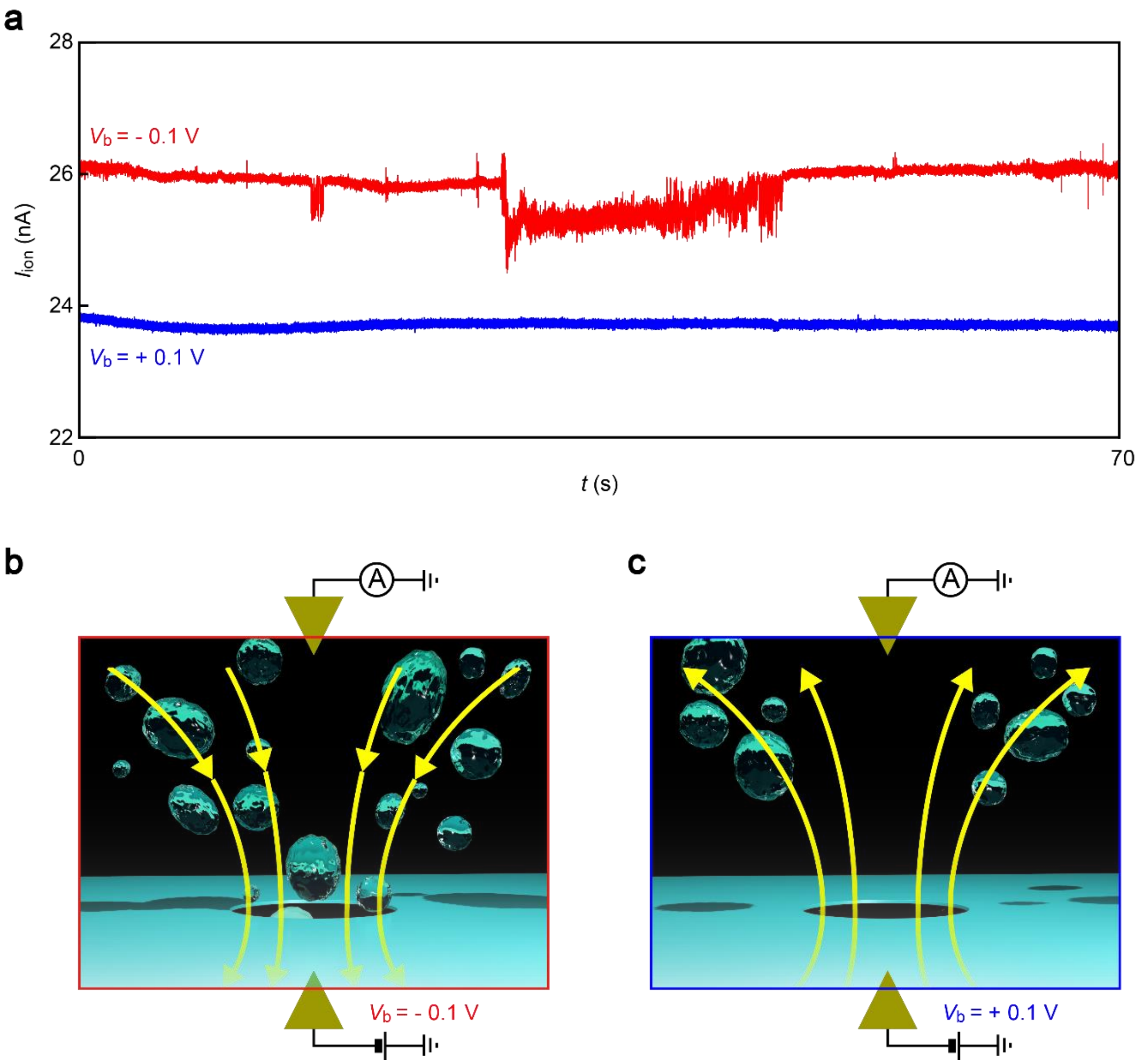

Figure S3. Electroosmotic filter. a, Ionic current traces recorded under the applied dc voltage of + 0.1 V (blue) and – 0.1 V (red) in a nanopore filled with chorioallantoic fluid from one side and 0.25 x PBS on the other side. **b,** When $V_b < 0$ V, chorioallantoic solution is flown into the nanopore due to the negative native charges on the wall surface as indicated by yellow arrows. When this is the case, the ionic current traces show large fluctuations indicating hydrodynamic capturing of any bioparticles of small surface charges existing in the chorioallantoic fluid. **c,** On the other hand, $V_b > 0$ V generates the flow in opposite direction that induces a flow of the PBS into the nanopore. This serves to impede the invasion of the bioparticles in chorioallantoic solution into the nanopore. As influenza viruses are negatively charged, this electroosmotic filter protects the pore surface from being contaminated by the ingredients in chorioallantoic fluid during the virus detections.

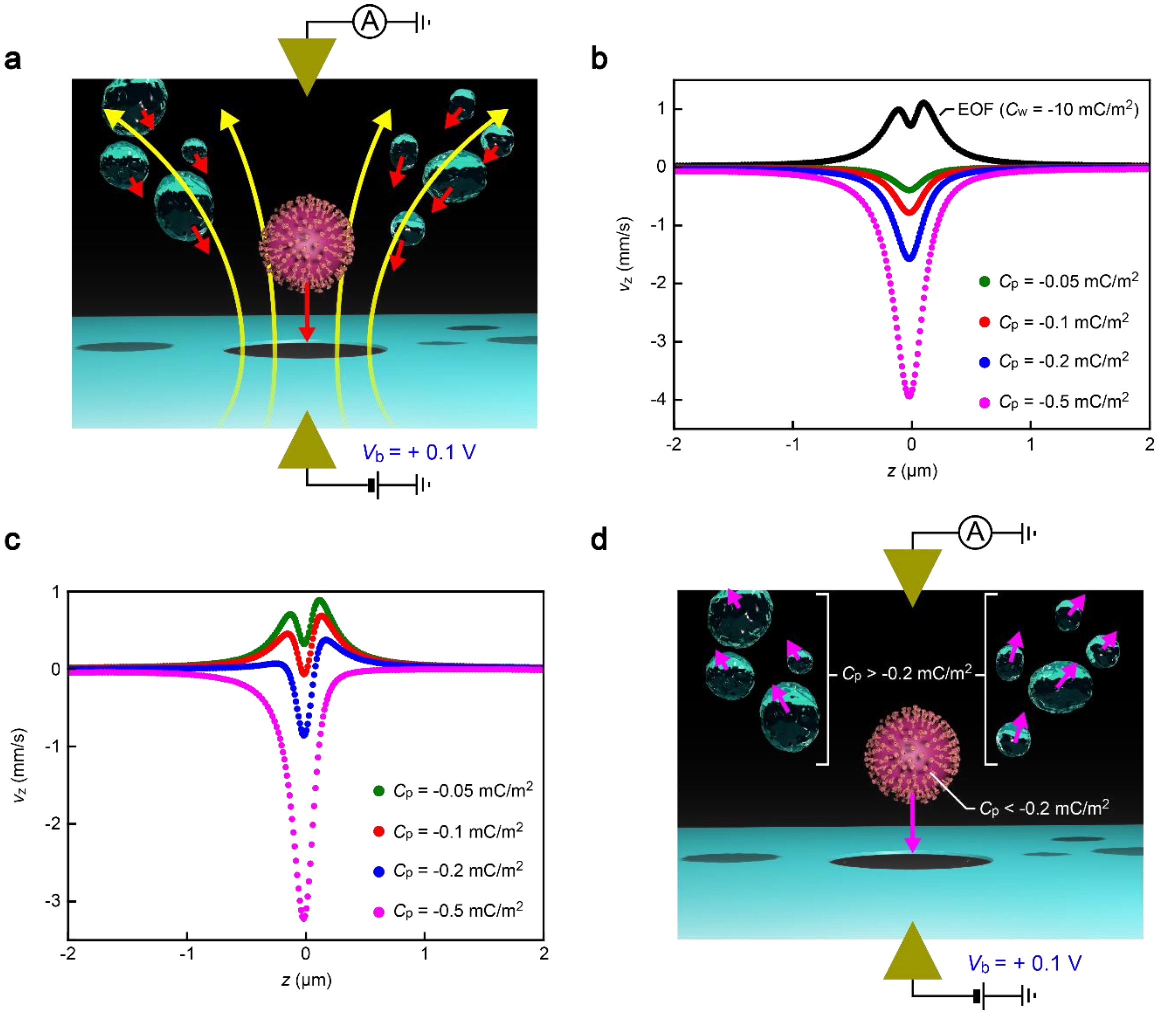

Figure S4. Particle charge dependence of the electroosmotic filter effects. a, Schematic illustration depicting the forces exerted on the influenza virus and the contaminants in chorioallantoic fluid. Yellow and red arrows describe the electroosmotic flow (EOF) and the electrophoretic forces under V_b = + 0.1 V. Here, the contaminants are considered as negatively charged. **b,** Particle velocity v_z along the pore axis under the electroosmotic (black) or electrophoretic force (other colors) alone calculated using COMSOL. v_z is positive (negative) when the particle is moved away from (toward) the nanopore. Due to the negative surface charges on the Si_3N_4 nanopore, EOF tends to flow in direction opposite to the electrophoresis of negatively-charged nanoparticles. Meanwhile, the electrophoresis become faster with increasing the amount of the particle surface charge C_p. **c,** v_z under the influence of EOF and the electrophoretic force. $v_z > 0$ mm/s at the pore orifice indicates blocking of the particles. On contrary, $v_z < 0$ mm/s in the entire z range simulated for C_p = - 0.5 mC/m^2 suggests electrophoretic translocation of the nanoparticles through the nanopore. **d,** As such, the nanoparticles of C_p > - 0.2 mC/m^2 are repelled at the orifice while only those of < - 0.5 mC/m^2 are allowed to enter and pass through the pore channel.

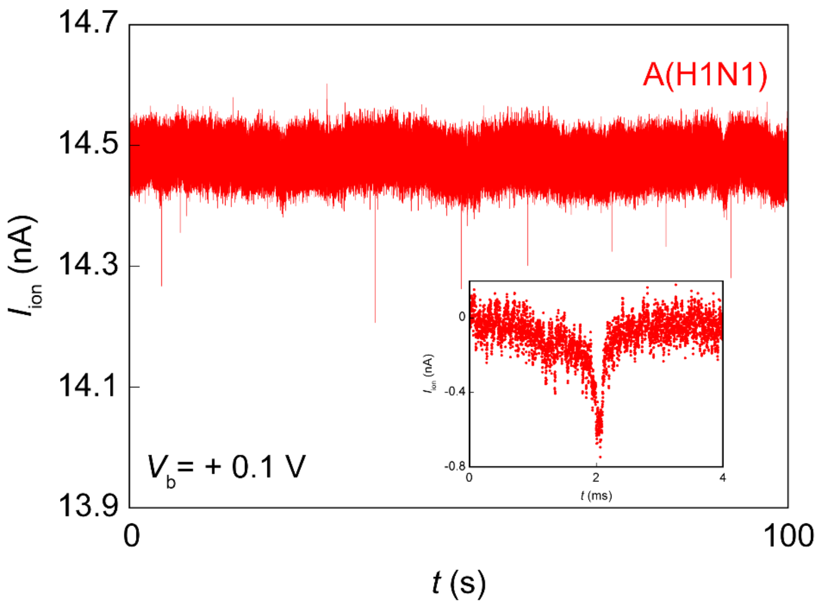

Figure S5. Influenza virus detections in human saliva. Ionic current trace obtained in human saliva (Lee Biosolutions, Inc.) containing influenza A (H1N1) using a Si_3N_4 nanopore of diameter 300 nm and length 50 nm under the V_b = + 0.1 V. The virus solution was prepared by first concentrating the virus via ultracentrifugal separation (86000 g, 45 min) and filtering of redispersion liquid at 450 nm after centrifugal separation (6000 rpm, 10 min) and filtering (450 nm) of the supernatant solution. The concentrated liquid was then mixed with the saliva at a volume rate of 5:50. Before the ionic current measurements, a small amount of 10 x PBS (4 %) was added to increase the ion concentration. Inset shows a magnified view of a resistive pulse recorded signifying electrophoretic translocation of the influenza virus through the nanopore.

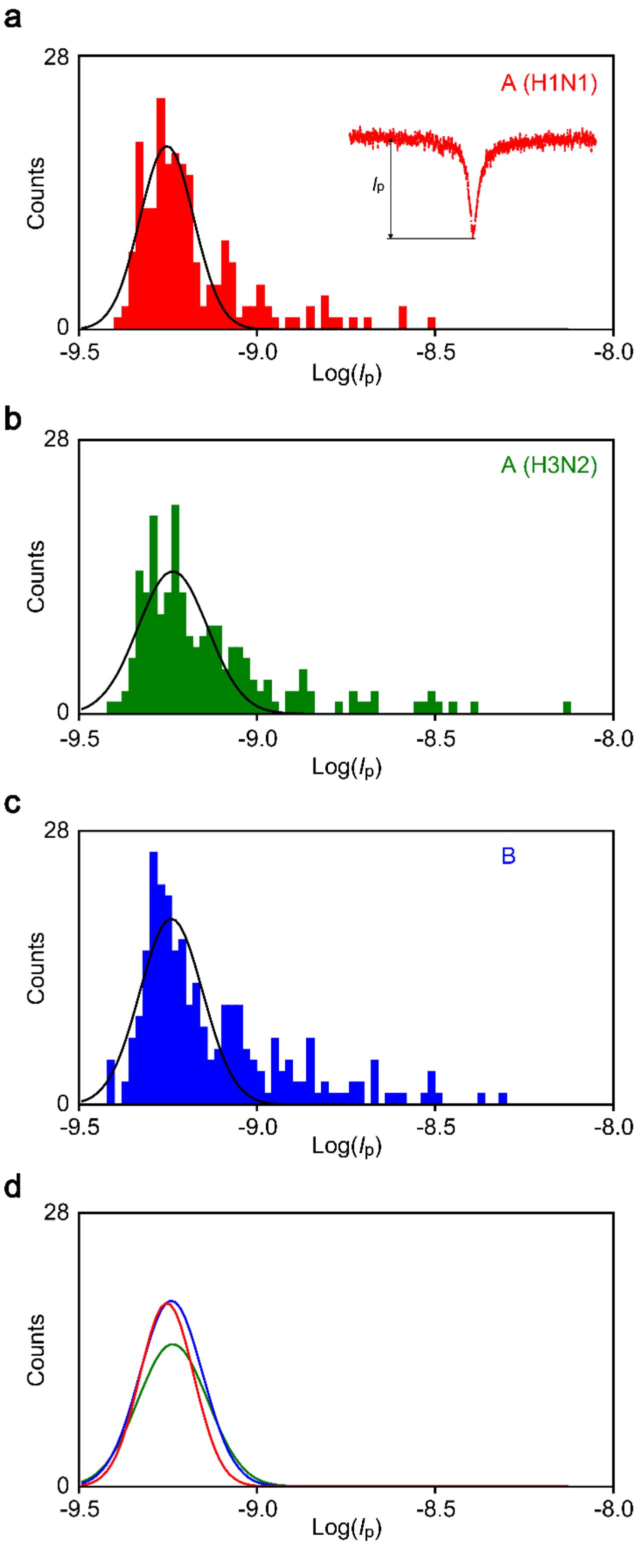

Figure S6. Spike height variations in influenza viruses. a-c, Histograms of the height of resistive pulses I_p obtained for A(H1N1) (a: red), A(H3N2) (b: green), and B (c: blue). Black curves are Gaussian fit to the distribution. **d,** Comparison of the I_p

distributions showing large overlap ascribed to the similar viral size among the types. Color coding is the same as that in (a-c).

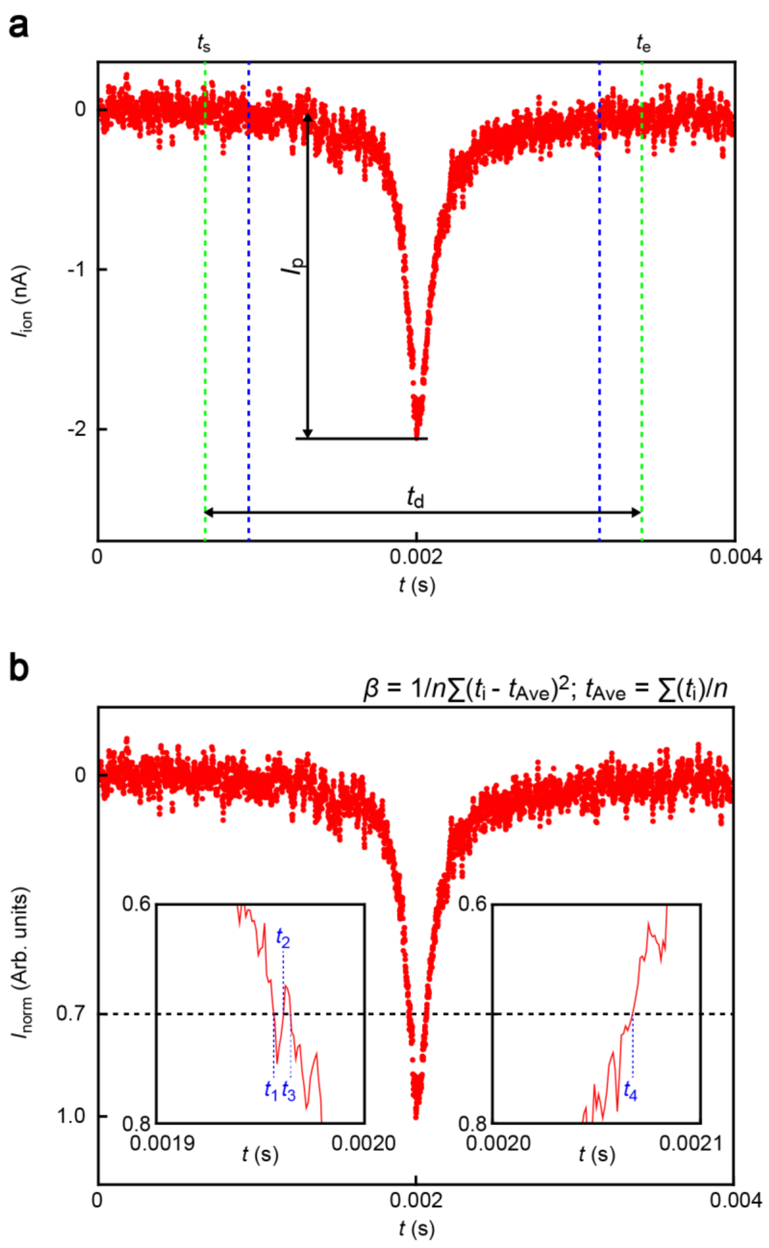

Figure S7. Definitions of pulse height, width, and bluntness. a, After offsetting the open pore current to zero, pulse onset was identified by the point where the ionic current fluctuation increased above 5σ (Blue dashed line at the left). In a similar way, the pulse offset was found as a point where the fluctuation decreased below 5σ (Blue dashed line at the right). The pulse region was then set by taking 0.256 ms margins at the both sides (green dashed lines), which defined the pulse width td as $t_d = t_e - t_s$, where t_e and t_s are the time stamp at the pulse onset and offset, respectively. In addition, the height of the pulse I_p was obtained from the current level at the pulse apex. **b,** Pulse bluntness β was defined as the variance of the data points where the pulses intersect the current level 70 % above I_p.

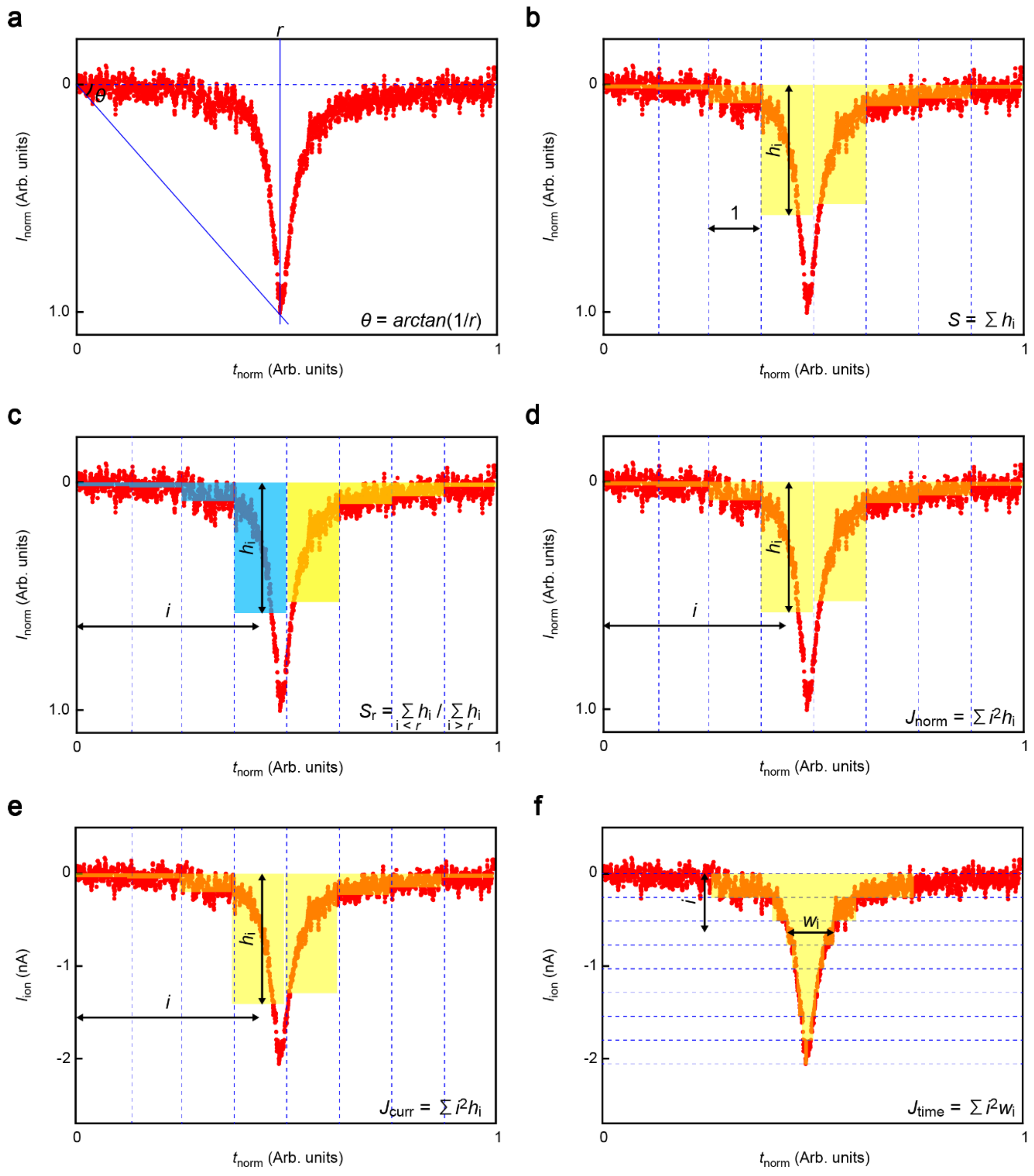

Figure S8. Definitions of other feature parameters. a, The onset angle θ was defined in the normalized pulses by I_p and t_d as $\theta = arctan(1/r)$, where r is the position of pulse apex. **b,** The pulse area S was calculated as a sum of the average pulse height h_i in the n-divided sections. **c,** The ratio S_r of the pulse area is obtained by dividing S of $i < r$ by that at $i > r$. **d,** The inertia J_{norm} with respect to the longitudinal axis in the normalized pulse. **e-f,** The inertia with respect to the longitudinal (e) and transverse axes (f) for the pulse normalized only with t_d.

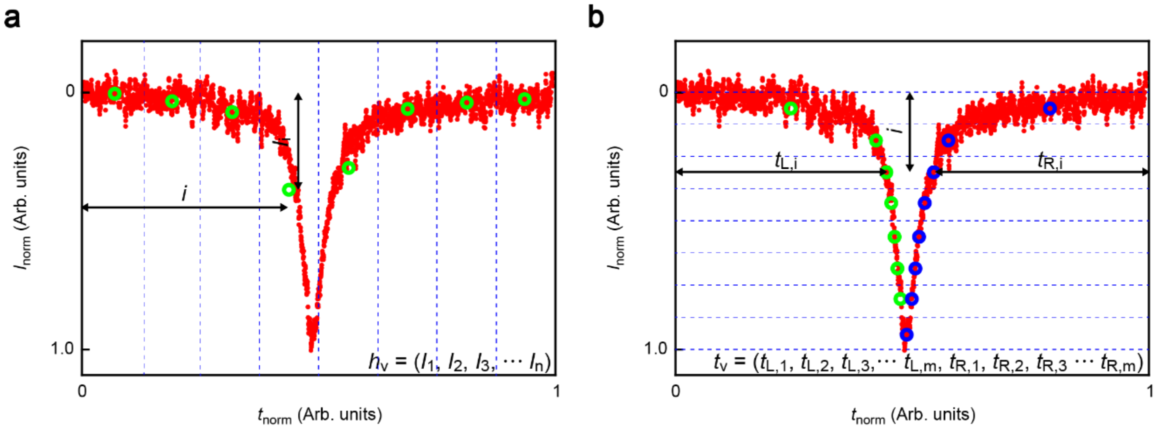

Figure S9. Definitions of current and time vectors. a, The current vector h_v was created as a sequence of average current levels in each of the *n*-divided sections of the normalized pulse. **b,** The time vector t_v was acquired as a sequence of the average time t_i in the *m*-divided sections of the normalized pulse. Here, we further classified the pulse region into the onset (green plots: $i < r$) and tail compartments (blue plots: $i > r$).

Table S1. Combinations of feature parameters and the number of feature vectors.

Feature parameters	*Number of feature vectors*
h_v (n = 8, 16, 32, 64, 128)	5
h_v (n = 8, 16, 32, 64, 128) & (I_p, t_d, β, θ, r, S, S_r, J_{time}, J_{curr}, J_{norm})	5
h_v (n = 8, 16, 32, 64, 128) & non-normarized t_v (m = 8, 16, 32, 64, 128)	25
h_v (n = 8, 16, 32, 64, 128) & t_v (m = 8, 16, 32, 64, 128)	25

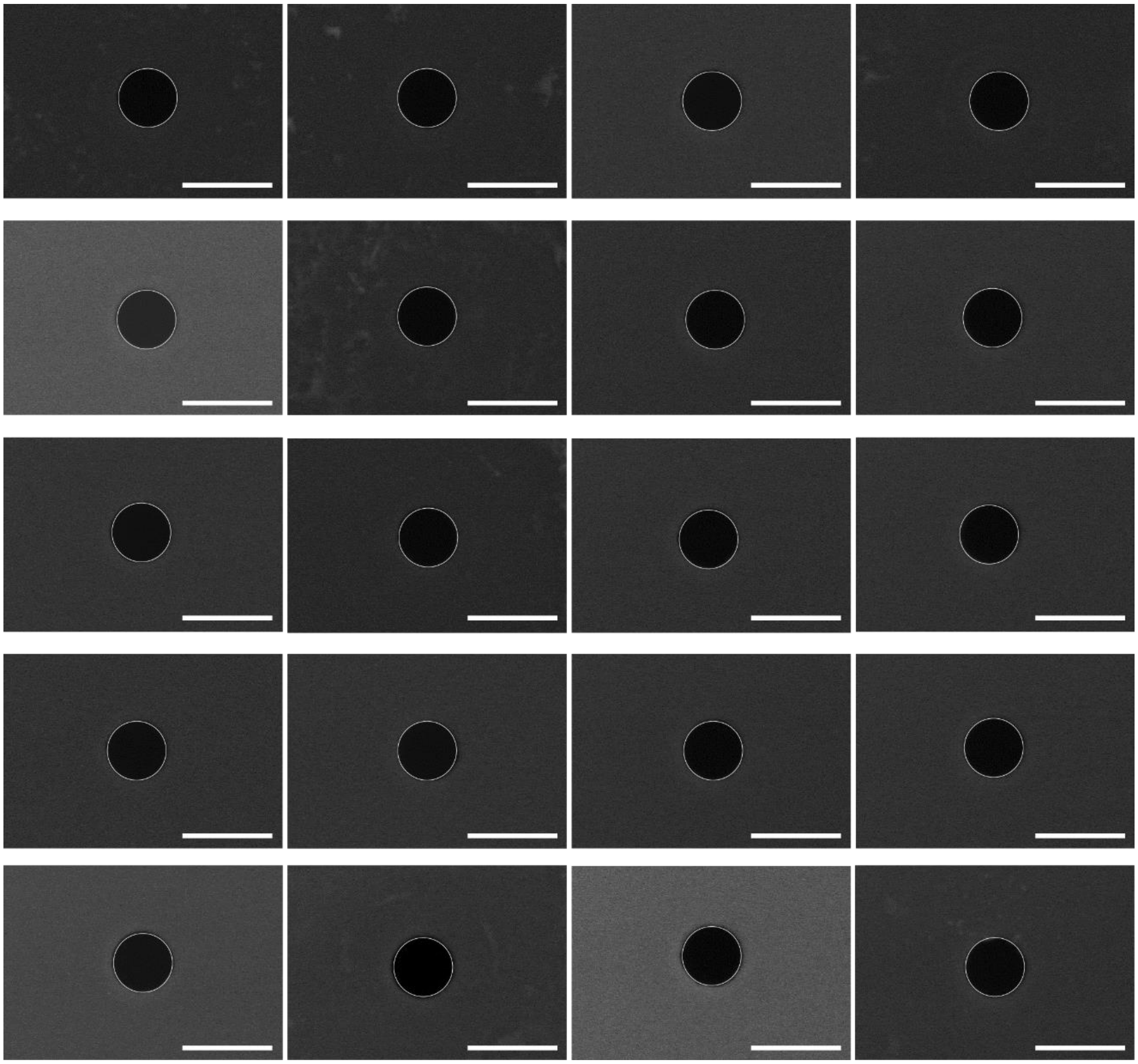

Figure S10. Variation in the nanopore size. Scanning electron micrographs of 20 nanopores fabricated on different Si chips. White circles indicate the 300 nm-sized pore. White bars denote 500 nm. The variation in the diameter is less than 5 % as assessed from the microscopy images.

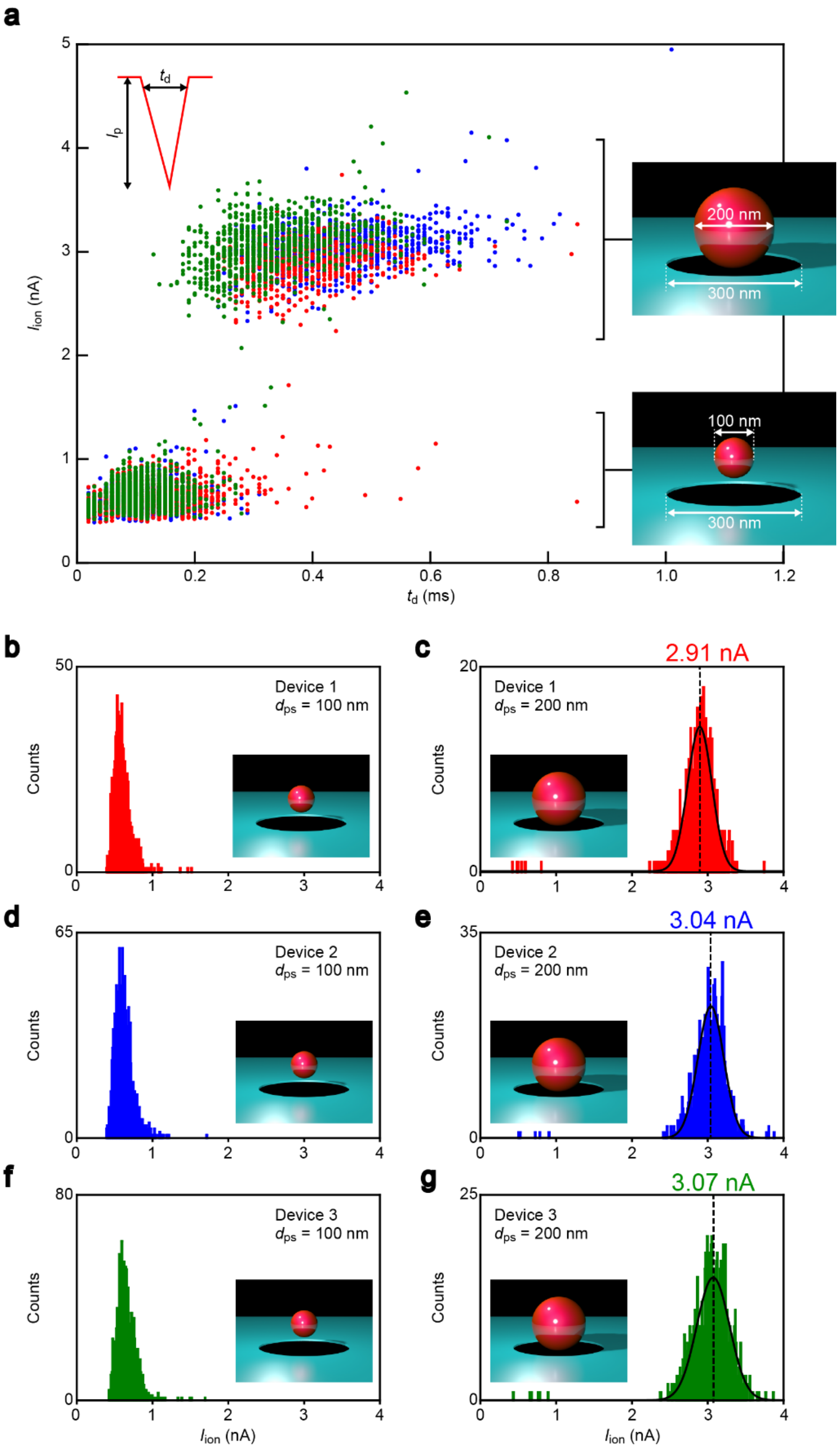

Figure S11. Reliability of the nanopore sensors. a, Scatter plots of the resistive pulse height I_p versus width t_d for the case of 200 nm- and 100 nm-sized carboxylated polystyrene nanobeads translocating through the 300 nm-sized nanopores. Color coding indicates the difference in the pores used, which were designed to have 300 nm

diameter but fabricated on different Si chips. Inset is a schematic explanation of I_p and t_d. Clusters of data below 1 nA and above 2 nA are attributed to the pulses obtained for the 100 nm- and 200 nm-sized nanoparticles, respectively. **b-g**, I_p histograms of the 100 nm- (b-d) and 200 nm-sized nanoparticles (e-g). Color code is the same as that in (a). The distributions show around 5 % variance in the pulse height, which is ascribed to the small variation in the channel size.

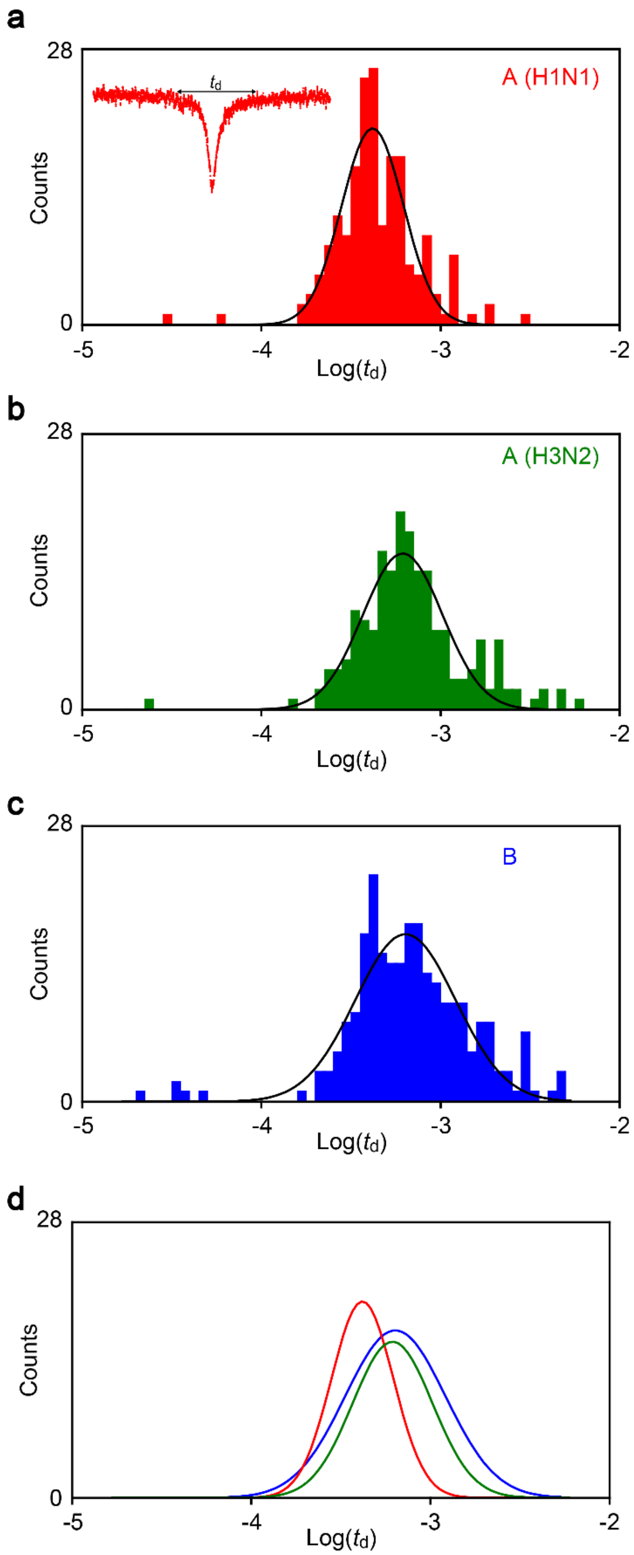

Figure S12. Spike width variations in influenza viruses. a-c, Histograms of the width of resistive pulses t_d obtained for A(H1N1) (a: red), A(H3N2) (b: green), and B (c: blue). Black curves are Gaussian fit to the distribution. **d,** Comparison of the t_d distributions showing relatively shorter pulse width for A(H1N1) than that of the other

two types, which suggests relatively fast electrophoresis of A(H1N1) viruses through the nanopore due presumably to the larger amount of negative surface charge on the viral surface compared to A(H3N2) and B. Color coding is the same as that in (a-c).

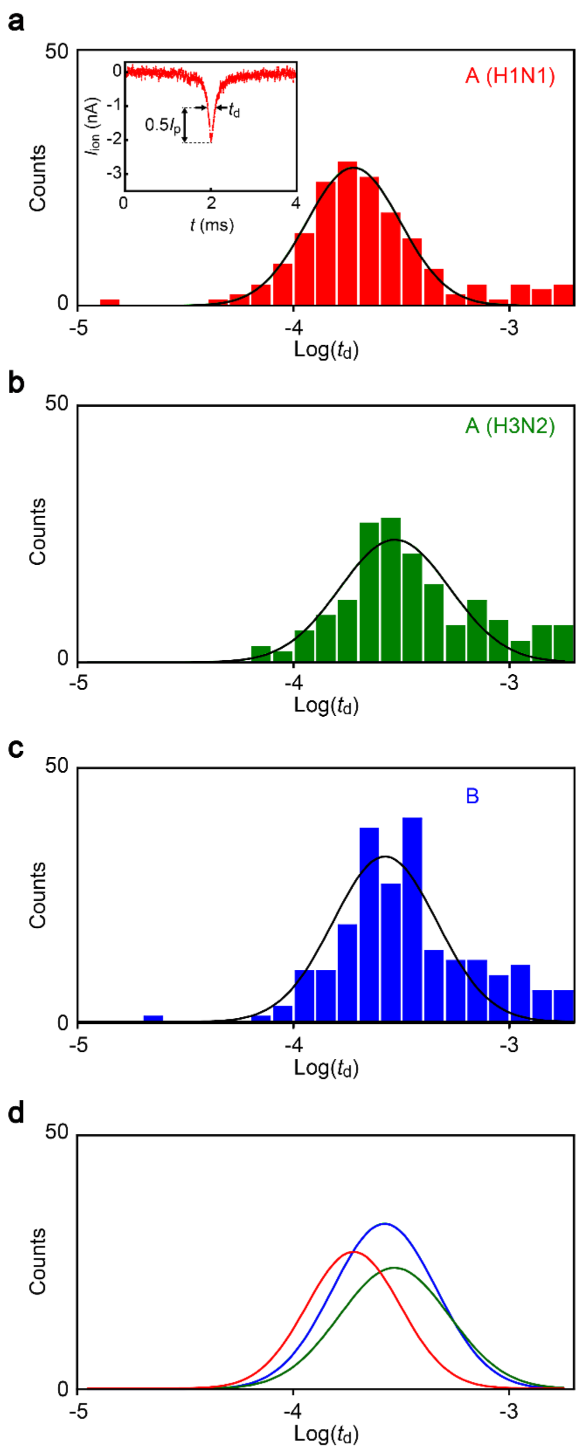

Figure S13. Influence of reference current levels to extract t_d. a-c, Histograms of the width of resistive pulses t_d at 0.5 I_p (inset) obtained for A(H1N1) (a: red), A(H3N2) (b: green), and B (c: blue) , where I_p is the pulse height. Black curves are Gaussian fit to the distribution. **d,** Comparison of the t_d distributions in (a) to (c) showing qualitatively the same order of difference in t_d among the three viruses. Color coding is the same

as that in (a-c). Whether this can give better virus discriminability via the machine learning-driven resistive pulse pattern analysis can be estimated from P_{rec} with the pulse bluntness β (Fig. 4 in the main text) that corresponds to the resistive pulse width at $0.3I_p$ from the apex. As P_{rec} is lower with β than that obtained with t_d, it is likely that the difference in the pulse width among the influenza viruses is smaller at the apexes.

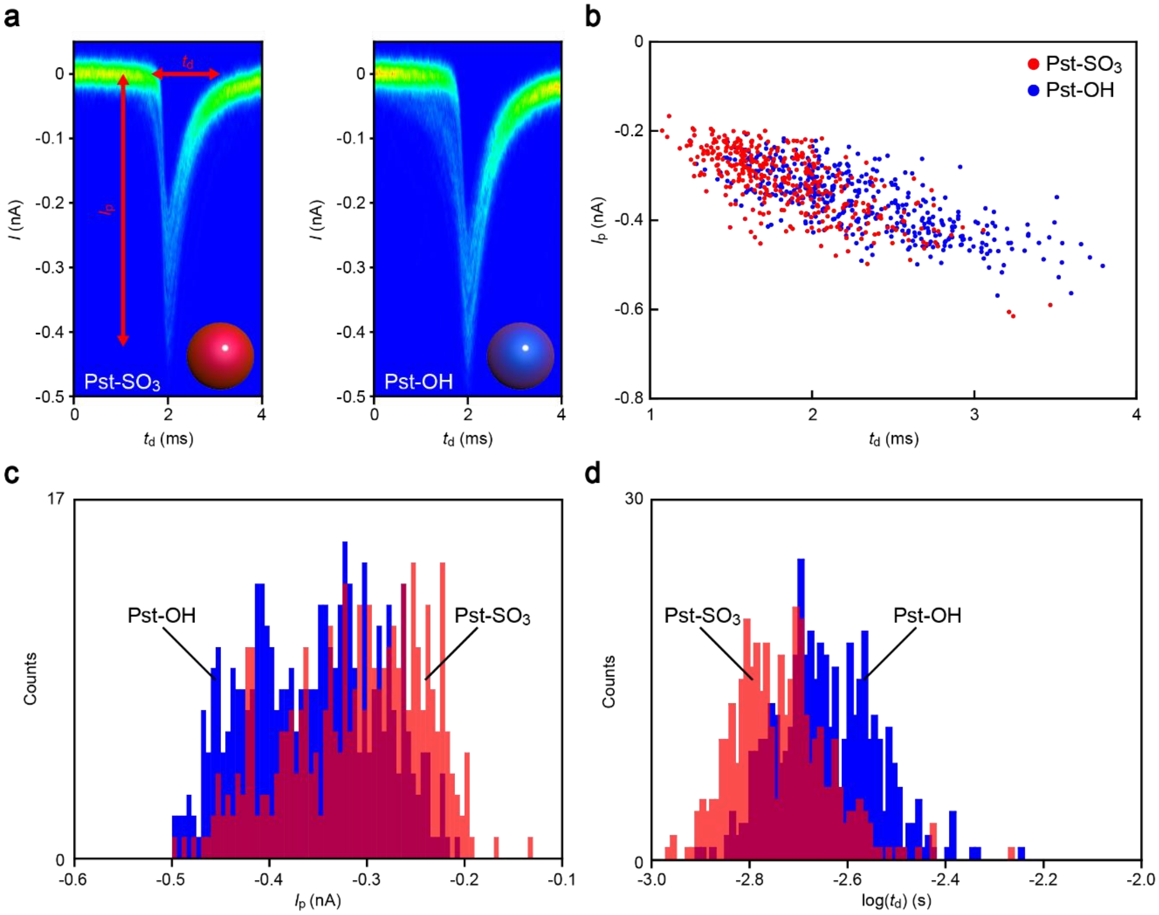

Figure S14. Ionic current signal characteristics of equi-sized Pst nanoparticles. a, Two-dimensional histograms of resistive pulses obtained for 500 nm-sized polystyrene (Pst) nanoparticles whose surface functionalized with SO_3 (left) and OH groups (right) with Si_3N_4 nanopore having diameter and thickness of 800 nm and 40 nm, respectively. **b,** The pulse height (I_p) versus width (t_d) scatter plots showing large overlap. **c,** I_p distributions of Pst-SO_3 (red) and Pst-OH (blue). While the extent of the variations is similar, the peak profiles are slightly different indicating a small difference in the size distributions between the nominally equi-sized nanoparticles. **d,** t_d distributions of Pst-SO_3 (red) and Pst-OH (blue). The shorter t_d of Pst-SO_3 indicates faster translocation speed of the nanoparticle through the nanopore, which is consistent with the smaller ζ potential of Pst-SO_3 (-86.8 mV) compared to that for Pst-OH (-75.6 mV) as measured using a zeta sizer.

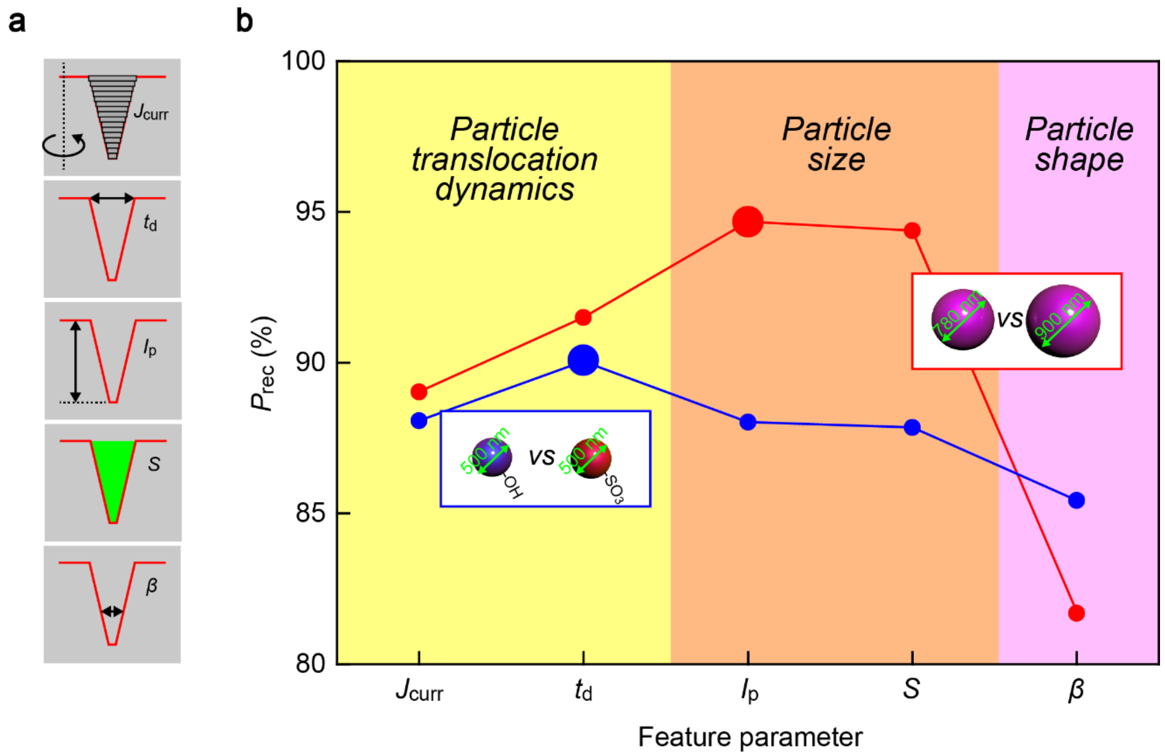

Figure S15. Discrimination of nanoparticles. a, Resistive pulse feature parameters used for the discrimination. **b,** Discriminability of the nanoparticles represented by the precision P_{rec} deduced using a non-parametric probability density estimation. Red and blue plots are P_{rec} calculated using a distinct feature parameter for discriminating Pst particles of different size (with COOH group on the surface) and equi-sized but different surface functionalization, respectively as illustrated in the insets. The highest P_{rec} is highlighted by lager circle illuminating the accordance of the feature parameter characteristics to the actual difference in the physical properties of the particles measured.

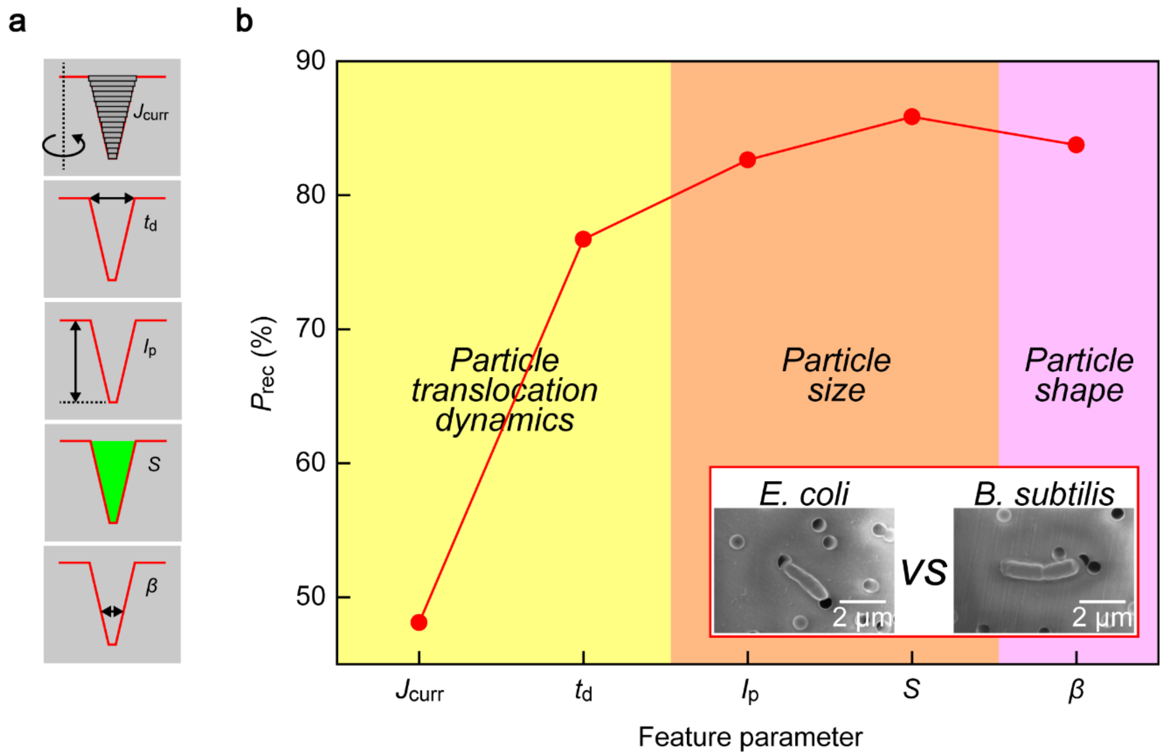

Figure S16. Discrimination of bacteria. a, Resistive pulse feature parameters used for the bacterial discrimination. **b,** Discriminability of two bacteria of similar size and surface charge states, *Escherichia coli* (*E. coli*) and *Bacillus subtilis* (*B. subtilis*), represented by the precision P_{rec} deduced using a non-parametric probability density estimation. Inset shows scanning electron microscopy images of the rod-like-shaped bacteria (single *E. coli* and two *B. subtilis*) of similar thickness and length. The feature-dependent P_{rec} show that there is little difference in the translocation dynamics of the bacteria, which is in accordance with the fact that they possess very similar ζ potentials of around -47 mV as measured with a zeta sizer. In contrast, the bacteria are more feasible to distinguish by their size and most notably the shapes. In fact, the microscopy observations confirm the rather rounded motifs of *E. coli* compared to the somewhat edged structure of *B. subtilis*.
